# Remote monitoring of implantable loop recorders reduces time to diagnosis in patients with unexplained syncope: a multicenter propensity score-matched study

**DOI:** 10.3389/fcvm.2023.1193805

**Published:** 2023-06-14

**Authors:** Vincenzo Russo, Anna Rago, Nicola Grimaldi, Raffaele Chianese, Aniello Viggiano, Giuliano D’Alterio, Diego Colonna, Agostino Mattera Iacono, Andrea Antonio Papa, Andrea Spadaro Guerra, Alessio Gargaro, Antonio Rapacciuolo, Berardo Sarubbi, Antonio D’Onofrio, Gerardo Nigro

**Affiliations:** ^1^ Cardiology and Syncope Unit, Department of Medical Translational Sciences, University of Campania “Luigi Vanvitelli”—Monaldi Hospital, Naples, Italy; ^2^ Adult Congenital Heart Disease Unit, Monaldi Hospital, Naples, Italy; ^3^ Cardiology Division, Sant'Anna and San Sebastiano Hospital, Caserta, Italy; ^4^ Department of Advanced Biomedical Sciences, Federico II University of Naples, Naples, Italy; ^5^ Cardiology Department, Electrophysiology and Cardiac Pacing Unit A.O.R.N. V. Monaldi, Naples, Italy; ^6^ Clinical Research Unit, Biotronik Italia S.p.A., Cologno, Italy

**Keywords:** syncope, implanted loop recorder, remote monitoring, arrhythmias, pacemaker

## Abstract

**Background:**

There are little data on remote monitoring (RM) of implantable loop recorders (ILRs) in patients with unexplained syncope and whether it confers enhanced diagnostic power.

**Objective:**

To evaluate the effect of RM in ILR recipients for unexplained syncope for early detection of clinically relevant arrhythmias by comparison with a historical cohort with no RM.

**Methods:**

SyncRM is a propensity score (PS)-matched study prospectively including 133 consecutive patients with unexplained syncope and ILR followed up by RM (RM-ON group). A historical cohort of 108 consecutive ILR patients with biannual in-hospital follow-up visits was used as control group (RM-OFF group). The primary endpoint was the time to the clinician's evaluation of clinically relevant arrhythmias (types 1, 2, and 4 of the ISSUE classification).

**Results:**

The primary endpoint of arrhythmia evaluation was reached in 38 patients (28.6%) of the RM-ON group after a median time of 46 days (interquartile range, 13–106) and in 22 patients (20.4%) of the RM-OFF group after 92 days (25–368). The PS-matched adjusted ratio of rates of arrhythmia evaluation was 2.53 (95% confidence interval, 1.32–4.86) in the RM-ON vs. RM-OFF group (*p* = 0.005).

**Conclusion:**

In our PS-matched comparison with a historical cohort, RM of ILR patients with unexplained syncope was associated with a 2.5-fold higher chance of evaluations of clinically relevant arrhythmias as compared with biannual in-office follow-up visits.

## Introduction

Implantable loop recorders (ILRs) are indicated in high-risk patients with unexplained syncope in whom comprehensive evaluation has failed to identify the cause of transient loss of consciousness and in those with suspected reflex syncope and frequent or severe syncopal recurrences (1). In the last decade, remote monitoring (RM) of cardiac implantable electronic devices has been consistently shown to safely reduce the burden of in-office visits while ensuring early detection of arrhythmias and adverse events (2). RM is now recommended as part of the standard follow-up management strategy (3). However, there are little data on the RM of ILRs in patients with unexplained syncope and whether it confers enhanced diagnostic power (4–6). Our study aimed to provide additional information about the effect of RM in this specific group of patients by comparing time to clinician's evaluation of clinically relevant arrhythmias in a prospective ILR remotely controlled cohort with a historical cohort conventionally followed with biannual in-hospital visits.

## Methods

### Study population and follow-up strategy

We conducted the SyncRM study, a prospective, multicenter, observational investigation including consecutive patients who received an ILR for unexplained syncope, between January 2019 and September 2021 at five investigational clinics (NCT04435262). Approvals from the local Ethics Committees were obtained for all participating sites. All these patients were remotely followed up (RM-ON group) with no prespecified schedule of in-office visits. A historical cohort of consecutive patients who had received an ILR for the same indication of unexplained syncope, between 2017 and 2019, was used as a control group (RM-OFF group), after balancing potential confounders with a propensity score (PS) method. The minimum study follow-up was 18 months.

Before undergoing ILR implantation for unexplained syncope and study enrollment, eligible patients had to complete the recommended syncope diagnostic workout, including clinical evaluation, electrocardiogram, echocardiogram, carotid sinus massage, and head-up tilt test (1).

All devices were implanted with standard techniques and programmed to detect atrial fibrillation with >12% RR interval variability, bradycardia episodes with <40 bpm heart rate, high ventricular heart rate episodes with >180 bpm, sudden rate drops >40%, and asystole episodes >3 s. All devices in the RM-ON group were remotely monitored with the Home Monitoring (HM) System (BIOTRONIK, Berlin, Germany) capable of daily transmissions of device data and arrhythmia-related diagnostics. Automatic RM alerts with notifications were activated only for the detection of episodes of asystole, bradycardia, sudden rate drop, and ventricular tachycardia. Detailed settings are reported in Table 1. Patients were not provided with the activator for manual electrocardiographic (ECG) self-recording in case of symptoms.

**Table 1:** Device programming and automatic remote monitoring alert setting.

Trigger type ms/bpm	Detection
AF	Low—12% RR variability
HVR	180 bpm
Bradycardia	40 bpm
SRD	40%
Asystole	3 s
Device	Type of alarm
Programmer triggered message received	Yellow warning
ERI	Red warning + notification
Backup mode active	Red warning + notification
Sensing	
Ventricular sensing amplitude (daily mean): <0.05 mV	Red warning + notification
Atr. and ven. arrhythmia	
Mean ventricular rate during AF > 150 bpm for >5% of day	Yellow warning
Number of asystole episodes: at least one	Red warning + notification
Number of high ventricular rate episodes (HVR): at least one	Red warning + notification
Number of bradycardia episodes: at least one	Yellow warning + notification
Number of sudden rate drop episodes: at least one	Yellow warning + notification
Episode	
Episode details received	Yellow warning
Remote monitoring	
First message received	Yellow warning
No messages received for 5 days	Yellow warning + notification
HM follow-up transmission has arrived	Yellow warning

AF, atrial fibrillation; HVR, high ventricular rate; SRD, sudden rate drop; ERI, elective replacement indicator; HM, home monitoring.

Patients or the public were not involved in the design, conduct, reporting, or dissemination plans of our research.

This study was approved by the Ethics Committee Università degli Studi della Campania “Luigi Vanvitelli”—A.O.U. “Luigi Vanvitelli”, A.O.R.N. “Ospedali dei Colli” ID 181060320. All methods were performed following the relevant guidelines and regulations, and informed consent was obtained from all subjects and/or their legal guardian(s).

The datasets used and/or analyzed during the current study are available from the corresponding author upon reasonable request.

### Study endpoints

The primary endpoint was the time to the clinician's evaluation of true relevant arrhythmias automatically detected by ILR. The following events, irrespective of symptoms, were considered [type 1, 2, and 4 of the ISSUE classification (7)]: Mobitz 2 s- or third-degree atrioventricular block, sinus arrest, bradycardia with <40 bpm heart rate for >10 s or >40% drop, and prolonged rapid paroxysmal supraventricular and ventricular tachycardia. False events, i.e., automatic registrations without arrhythmic events (artifacts), were assessed and filtered out by the investigators. All events were adjudicated by two blinded investigators (VR and RC) with a third opinion in cases of disagreement (AD'O). We further reported the time to evaluation of asymptomatic true ILR-detected episodes and of arrhythmias eventually leading to pacemaker implantation.

To control bias in comparisons, the two study groups were balanced with respect to 30 potential confounding variables (including demographics, cardiovascular diseases, history of syncope, arrhythmias, and therapy), using the propensity score as described below.

### Statistical analysis

Continuous variables were reported as mean ± standard deviation if normally distributed, or as median (interquartile range) if non-normally distributed. Normality was tested with the Shapiro–Wilk method. Binary or categorical variables were reported as count (percentage of non-missing values). Comparisons of continuous variables were performed with the Student’s *t* test or Mann–Whitney *U* test depending on the result of the normality test. Categorical variables were compared with Pearson's Chi-squared test or Fisher exact test, when appropriate.

Distributions of times to event evaluations were plotted with the Kaplan–Meier method and the hazard ratios (HRs) in the RM-ON vs. RM-OFF groups were reported as a measure of ratios of the rate of event evaluations between study groups.

Survival analysis was performed for primary and secondary endpoint events, computing event-free rates by study groups (RM-ON vs. RM-OFF) using uni- and multivariate Cox proportional hazard models. To reduce uncontrolled biases due to the nonrandomized study design, we used the PS to match the study groups with the inverse probability of the treatment weighting method (8). Balancing weights were based on the “average treatment effect in the treated” estimand, generated by generalized boosted models [a machine learning technique (9)]. The selection of covariates for PS computing was based on the following criteria: variables thought to be related to the treatment assignment or outcome; imbalanced variables as revealed by significant comparisons between treated and control groups; variables with <15% missing data. Balancing of covariates was considered successful if the relative between-group standardized difference was ≤0.10 in each covariate after PS matching. Residual unbalanced covariates were used as adjusting variables in survival models. Standard unmatched analyses were also performed for reference. Adjusted and unadjusted HRs and relative 95% confidence interval (CI) were reported.

Analyses were performed with R software version 4.1.0 (R Foundation for Statistical Computing, Vienna, Austria). Propensity scores were calculated with the WeightIt package (version 0.12.0).

## Results

### Study population

A total of 241 patients (54 ± 22 years; 35.3% male) were included in the study; 133 (55.2%) were in the RM-ON group and 108 (44.8%) were in the RM-OFF group. The clinical characteristics of the study population are summarized in Table 2. Patient groups did not differ significantly in most variables. As compared to the RM-OFF group, patients in the RM-ON group were younger (54 vs. 64 years median age, *p* = 0.007).

**Table 2:** Patient characteristics.

Characteristic	RM-ON, *N* = 133^a^	RM-OFF, *N* = 108^a^	*p*-value^b^
Age	54 (28, 71)	64 (48, 76)	0.007
Male	91 (68%)	65 (60%)	0.2
Smoke	33 (26%)	21 (23%)	0.6
Familiarity of sudden death	17 (13%)	12 (13%)	0.9
History of arrhythmia	19 (15%)	18 (19%)	0.4
Hypertension	58 (45%)	38 (41%)	0.5
Dyslipidemia	35 (27%)	20 (22%)	0.3
Coronary artery disease	16 (12%)	11 (12%)	0.9
Chronic obstructive pulmonary disease	12 (9.3%)	17 (18%)	0.05
Diabetes	10 (7.8%)	12 (13%)	0.2
Previous stroke or TIA	5 (3.9%)	5 (5.4%)	0.7
Atrial fibrillation	14 (11%)	14 (15%)	0.4
Chronic kidney disease	8 (6.2%)	8 (8.6%)	0.5
Palpitations	29 (22%)	22 (24%)	0.8
History of syncope	133 (100%)	108 (100%)	
Total number of syncope	2 (1, 4)	3 (2, 4)	0.06
Prodromes	57 (43%)	30 (33%)	0.14
Presyncope	39 (29%)	24 (26%)	0.6
Trauma	36 (27%)	23 (26%)	0.8
Resting heart rate	70 (62, 80)	70 (65, 75)	0.6
Systolic blood pressure	120 (111, 130)	120 (115, 128)	0.9
Left ventricle ejection fraction (%)	59 (55, 60)	55 (55, 60)	0.5
Medical therapy			
ACE inhibitors	32 (25%)	22 (22%)	0.6
Angiotensin receptor blockers	14 (11%)	10 (9.9%)	0.8
Beta blockers	35 (28%)	30 (30%)	0.7
Diuretics	15 (12%)	10 (9.9%)	0.6
Calcium antagonists	16 (13%)	8 (7.9%)	0.3
Antiplatelets	22 (17%)	20 (20%)	0.6
Anticoagulants	12 (9.4%)	2 (2.0%)	0.020
Antiarrhythmic class IC agents	6 (4.7%)	5 (5.0%)	0.9
Amiodarone	3 (2.4%)	1 (1.0%)	0.6
Sotalol	5 (3.9%)	2 (2.0%)	0.5
Insulin	19 (15%)	16 (16%)	0.9

ACE, angiotensin-converting enzyme; TIA, transient ischemic attack.

^a^ Median (interquartile range) or *n* (%).

^b^ Wilcoxon rank sum test, Pearson's Chi-squared test, or Fisher's exact test.

**Figure 1:**
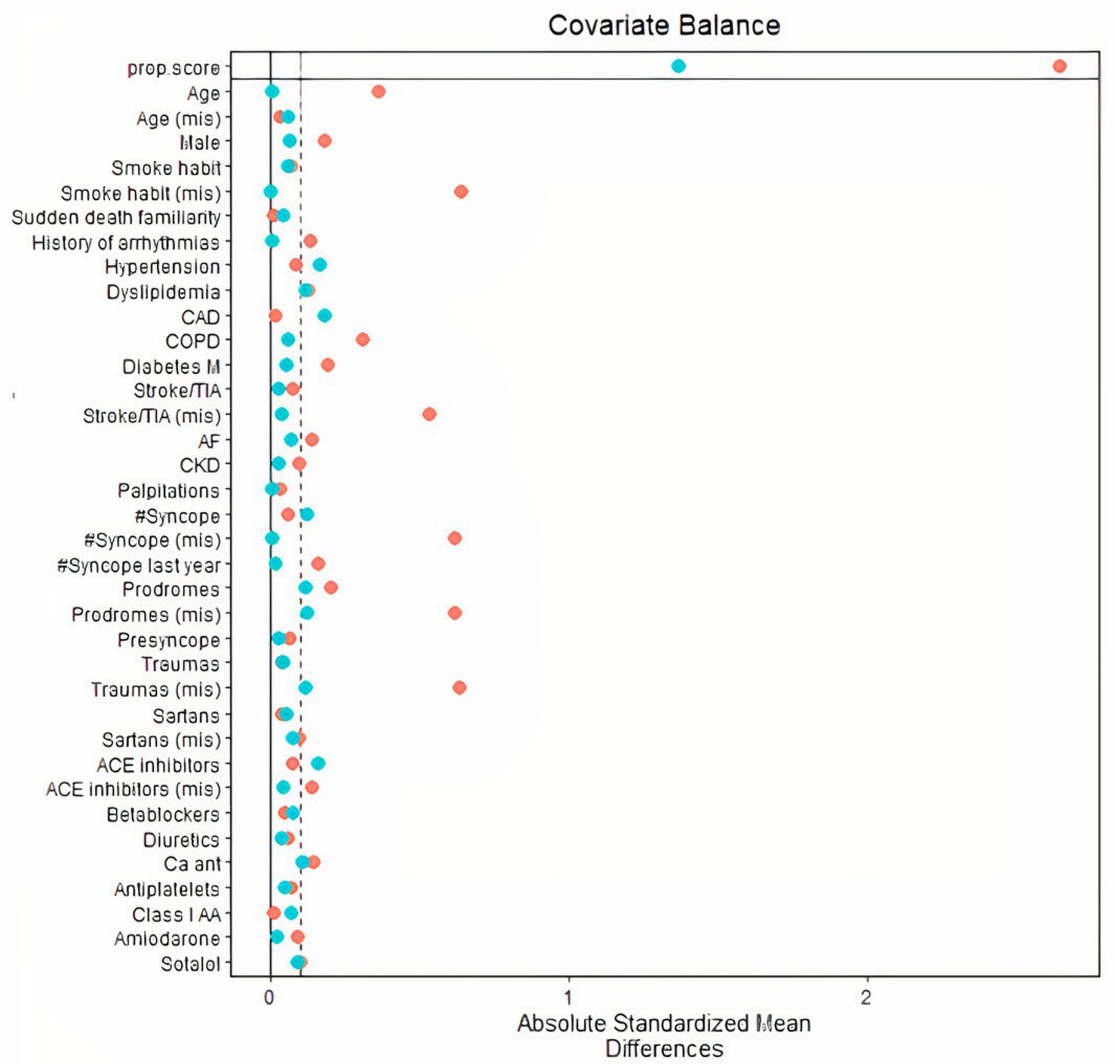
Distribution of absolute standardized mean differences across 30 population variables (including demographics, cardiovascular diseases, history of syncope and arrhythmias, therapy) before inverse probability of treatment weighting PS matching (red dots), and after PS matching (blue dots). After PS matching, all variables were sufficiently balanced as compared to original unmatched variables. CAD and hypertension were associated to standardized differences markedly exceeding 0.10 (dashed vertical line) and were used as adjusting covariates in survival models. PS, propensity score; CAD, coronary artery disease.

However, after PS matching, absolute standardized mean difference of all the 30 variables was reduced to 10% or less (Figure 1), except for coronary artery disease and hypertension. These two variables were further included as adjusting covariates in subsequent multivariate models.

### Study endpoints

During a median follow-up of 22 (12–34) months, arrhythmias were detected in 38 patients (28.6%) in the RM-ON group and in 22 patients (20.4%) in the RM-OFF group. Overall, 46 events (76.7%) were sinus bradycardias/arrests or atrioventricular blocks according to types 1 and 2 of the ISSUE classification. In the RM-ON group, post-diagnosis medical interventions were pacemaker implantations in 25 patients (18.8%), supraventricular tachycardia ablation in 1 patient (0.8%), and therapy change/adjustment in 3 patients (2.2%). In the RM-OFF group, there were 14 pacemaker implantations (13.0%), 2 supraventricular tachycardia ablations (1.8%), and 6 therapy change/adjustments (5.5%). Conservative measures, including hydration, salt consumption, reducing the hypotensive agents, and recognition of prodromal symptoms, were always recommended in patients with suspected typical reflex syncope. Pacemaker implantation was considered after screening according to age (>40 years) and duration of the ILR-recorded asystole recorded (>3 s symptomatic, >6 s asymptomatic), as suggested by the guidelines.

ILR-detected events eliciting diagnosis were asymptomatic in 24 patients (18.0%) in the RM-ON group and in 8 (7.4%) in the RM-OFF group, eventually leading to pacemaker implantation in 12 (9.0%) in the RM-ON group and in 2 (1.9%) in the RM-OFF group (Table 3).

**Table 3:** ILR-detected events, symptoms, and medical interventions.

	RM-ON, *N* = 133	RM-OFF, *N* = 108
ILR-detected events		
Sinus arrest/bradycardia	13 (9.8%)	5 (4.6%)
AV block	19 (13.3%)	9 (8.3%)
Atrial fibrillation	4 (3.0%)	5 (4.6%)
PSVT	1 (0.75%)	3 (2.8%)
VT	1 (0.75%)	0 (0.0%)
Asymptomatic	24 (18.0%)	8 (7.4%)
Medical interventions		
Pacemaker implantation	25 (18.8%)	14 (13.0%)
PSVT ablation	1 (0.8%)	2 (1.8%)
AADs initiation	1 (0.7%)	1 (0.9%)
AADs adjustment	2 (1.5%)	3 (2.8%)
OACs initiation	0 (0%)	2 (1.8%)

AV: atrio-ventricular; ILR: implantable loop recorder; PSVT: paroxysmal supraventricular tachycardia; RM: remote monitoring; VT: ventricular tachycardia; AADs: antiarrhythmic drugs; OACs: oral anticoagulants

**Figure 2:**
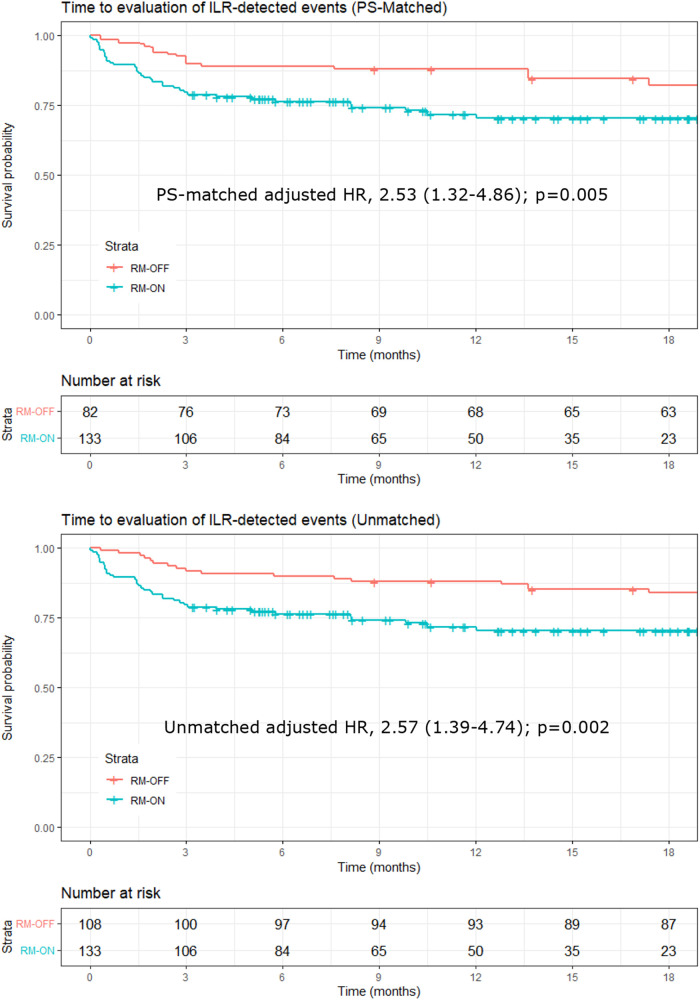
Kaplan–Meier plots of time to evaluation of ILR-detected events. Upper panel: PS-matched analysis; lower panel: unmatched analysis. In the PS-matched analysis, the number at risk in the RM-OFF group is reduced because of the weighting process of PS matching. ILR, implantable loop recorder; PS, propensity score.

Ratios of event evaluation rates between groups are reported in Table 4 in terms of adjusted and unadjusted HRs. The PS-matched (adjusted) HR was 2.53 (CI, 1.32–4.86) in the RM-ON vs. RM-OFF group (*p* = 0.005), reflecting a 2.5-fold higher rate of event evaluations in the RM-ON group as compared to the RM-OFF group (Figure 2). The median time to event evaluations was 46 days (interquartile range, 13–106) in the RM-ON group and 92 days (25–368) in the RM-OFF group.

**Table 4:** Ratios of rate of event evaluations in the RM-ON group vs. the RM-OFF group, expressed in terms of PS-matched, adjusted/unadjusted hazard ratios between study groups.

Variable	PS-matched		Unmatched
	Univariate HR (CI); *p*	Multivariate HR (CI); *p*	Multivariate HR (CI); *p*
All events			
Remote monitoring	2.22 (1.15–4.30); *p* = 0.017	2.53 (1.32–4.86); *p* = 0.005	2.57 (1.39–4.74); *p* = 0.002
Adjusting variables			
CAD		0.54 (0.21–1.41); *p* = 0.43	0.82 (0.38–1.76); *p* = 0.60
Hypertension		3.81 (2.09–6.94); *p* < 0.0001	2.89 (1.64–5.10); *p* = 0.0002
Asymptomatic events			
Remote monitoring	4.58 (1.64–12.76); *p* = 0.0003	4.28 (1.52–11.96); *p* = 0.006	4.58 (1.78–11.78); *p* = 0.002
Adjusting variables			
CAD		0.64 (0.19–2.12); *p* = 0.47	1.05 (0.39–2.86); *p* = 0.60
Hypertension		2.55 (1.15–5.64); *p* = 0.02	1.94 (0.93–4.22); *p* = 0.07
Events leading to pacemaker implantation			
Remote monitoring	2.18 (0.89–5.33); *p* = 0.08	2.82 (1.77–6.77); *p* = 0.02	2.31 (1.08–4.95); *p* = 0.03
Adjusting variables			
CAD		0.75 (0.29–1.98); *p* = 0.57	0.50 (0.16–1.57); *p* = 0.23
Hypertension		3.13 (1.51–6.46); *p* = 0.002	4.32 (1.99–9.38); *p* = 0.0002

CAD, coronary artery disease; CI, confidence interval; HR, hazard ratio; PS, propensity score; RM, remote monitoring.

**Figure 3:**
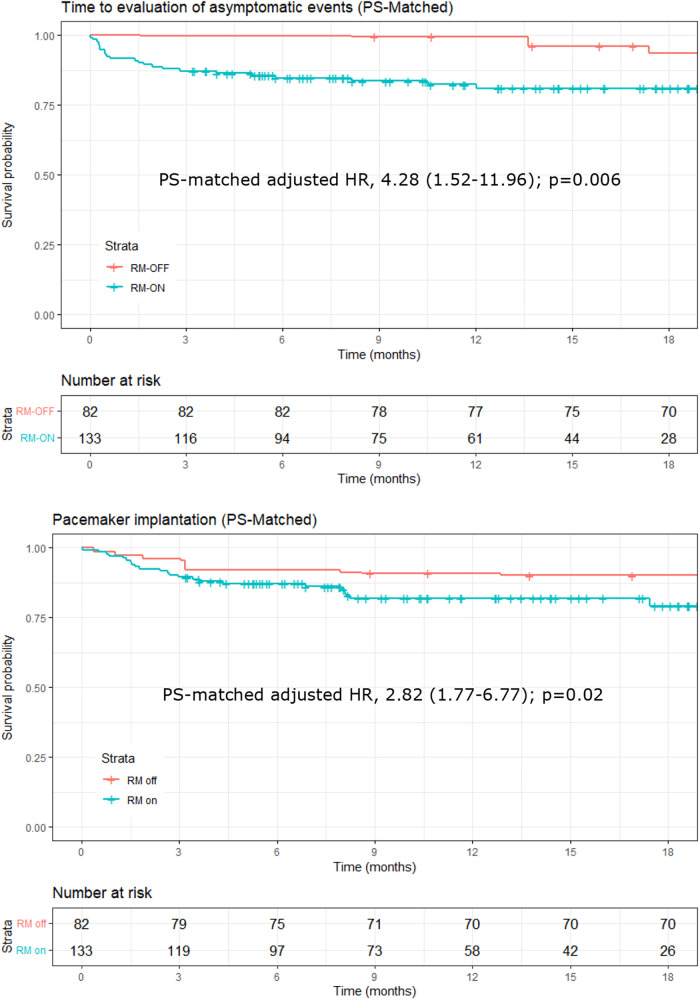
Kaplan–Meier plots of time to evaluation of asymptomatic events detected by the ILR (upper panel) and of events eventually leading to pacemaker implantation (lower panel). Plots and hazard ratios are PS-matched, and the number at risk in the RM-OFF group is reduced because of the weighting process of PS matching. ILR, implantable loop recorder; PS, propensity score.

When including only the 32 asymptomatic events in the analysis, the ratio of the rates of event evaluations almost doubled, with a PS-matched adjusted HR of 4.28 (CI, 1.52–11.96; *p* = 0.006) in the RM-ON vs. RM-OFF group (Figure 3, upper panel), and a median time to evaluation of 35 days (interquartile range, 7–119) in the RM-ON group and 240 days (83–470) in the RM-OFF group.

Finally, for ILR-detected events eventually leading to pacemaker implantation, the PS-matched adjusted HR was 2.82 (CI, 1.77–6.77; *p* = 0.02), reflecting a significantly shorter time in the RM-ON group [median 54 days (14–83)] as compared to the RM-OFF [median 89 days (32–306)] (Figure 3, lower panel).

### Post-intervention follow-up data

Post-intervention follow-up data for an additional 13 (7–16) months were available in the 29 patients of the RM-ON group who reached the study endpoint and received treatment. Of this subgroup, two patients of this subgroup (7%) experienced syncopal recurrence: one had received a pacemaker for symptomatic asystole; one had received a pacemaker for asymptomatic atrioventricular block.

## Discussion

In our PS-matched comparison of remotely monitored patients who received an ILR for unexplained syncope with a historical cohort of non-remotely monitored patients, RM enhanced the diagnostic power of ILR with a 2.5-fold higher probability of clinician's evaluation of arrhythmic episodes and a median 46 days earlier diagnosis vs. a biannual schedule of in-office visits (50% relative reduction). Our estimations were obtained by balancing the two groups with the PS-match method based on the inverse probability of treatment weighting (8). The method assigns PS-dependent weights to controls (rather than excluding individuals not fulfilling matching criteria). The method was effective in covariate balancing (Figure 1), despite retaining all individuals in the analysis reducing the loss of information.

The RM effect was almost double if events were asymptomatic (>4-fold higher chance of clinician's evaluation) with about 85% reduction in median time to diagnosis (35 vs. 240 days). The chance of a diagnosis of events leading to the indication of pacemaker implantation was also 2.8-fold higher with RM-ON vs. RM-OFF.

Syncope is a common medical problem with a frequency between 15% and 39% in the general population and an annual number of episodes of 18.1 to 39.7 per 1,000 patients (10). Reflex syncope is by far the most common cause (11) but determining the cause of recurrent unexplained syncope remains a clinical challenge and a permanent quest. ILRs are effective tools for the diagnosis of unexplained syncope and their diagnostic yield may be increased by RM for at least two reasons: first, RM enables immediate notification to clinicians of relevant arrhythmic events with automatic alerts; second, RM ensures virtually unlimited storage capacity of diagnostic information and ECG recording, avoiding episode overwriting in saturated device memories. Nevertheless, although the benefit of RM in pacemakers and implantable defibrillators has been extensively assessed, few studies have investigated the effect of RM in the ILR-based diagnosis of syncope (4–6).

In one observational study using weekly remote transmissions from ILR in patients with unexplained syncope (4), RM allowed the detection of relevant ECG records in two-thirds of patients after a median time of 11 days from ILR implant, allowing an estimation of a 71-day reduction of time to diagnosis as compared to an assumed scheme of quarterly in-office device interrogations. However, there was no control group, and the reduction could only be estimated. Also, events of symptoms without significant ECG modifications were reported in >50% of patients. Conversely, we only included ILR-detected arrhythmias and still obtained a 2.5-fold higher chance of diagnosis and a 50% reduction in time to event evaluations by using RM over a biannual schedule of in-office visits (from 92 to 46 days). A similar relative reduction of time to diagnosis was obtained for evaluations of relevant arrhythmic events (sinus arrests/bradycardias and atrioventricular blocks) eventually leading to pacemaker implantation. Finally, the RM effect was larger in asymptomatic events, as expected, where the chance of diagnosis was >4-fold higher.

Fast diagnosis is important for several reasons, including early therapy initiation, safety, and quality of life improvement. Moreover, it also helps reduce the duration of long-term monitoring and the need for device replacements while waiting for the diagnosis. Diagnostic rates have been reported to be 43% still after 2 years, and 26% of all achieved diagnoses occurred after 18 months from implantation (12). A 2.5-fold higher chance of diagnosis over a median 22-month follow-up may have obvious implications for care quality and related costs.

A 50% reduction in time to event evaluation is not unexpected with RM. With accurate alert programming and daily remote transmissions, RM enables immediate (within 24 h) notification of relevant episodes. Therefore, assuming a uniform distribution of events over time, the expected average time to diagnosis using RM should be about 50% of the between-visit interval of an in-office visit program. The device and alert programming we used should cover a wide range of arrhythmic events potentially underlying syncope (bradycardia, asystole, sudden rate drop, tachyarrhythmias) and may be easily replicated in any available device and RM system. However, the improvement of the diagnostic power of ILR conferred by RM may significantly depend on compliance and reliability of remote transmissions. In our study, we used an RM technology known to ensure daily transmissions with about 90% rates of successful transmissions (13, 14), and our results may not be directly extrapolated to other RM systems. It has been reported that the chance of device memory saturation increases significantly with intervals between RM transmissions and may require adjustments on an individual basis (4). Our main objective was to estimate the RM effect on time to diagnosis of true relevant arrhythmias. Therefore, we did not track false-positive episodes systematically nor did we estimate the increased burden of their evaluations, as this is mostly related to the technical limitations of implanted devices rather than to RM. However, RM may amplify the effects of false-positives via automatic notifications as it has been reported that only 15%–54% of the ILR-detected episodes are true-positives (4–6, 12–15), so artificial intelligence techniques may be useful to increase positive predictive value at least for atrial fibrillation detection (15). However, our data showed that despite the false-positives, RM is associated with a significantly faster diagnosis of arrhythmias underlying unexplained syncope.

## Limitations

Our study has several limitations, including the lack of randomization, the relatively small sample, and the use of a historical cohort as control group. In the RM-ON group, 25 of 32 patients with sinus arrest, >10 s bradycardia, or atrioventricular block underwent pacemaker implantation according to current guidelines. Many of them did not report symptoms in relation to the RM-detected arrhythmias, so the direct relation of clinical syncope with these arrhythmias could not be demonstrated. The clinical relevance of subclinical ILR-detected arrhythmias has been recently questioned in patients with cryptogenic stroke by the results of a subanalysis of the Loop Recorder Detection of Atrial Fibrillation to Prevent Stroke (LOOP) study, showing that the risk of syncope and sudden death was similar in patients with and without ILR, despite the significantly higher incidence of bradyarrhythmias detected in the ILR group (16). However, our study cohort was substantially different, with clinical presentation of recurrent unexplained syncope and ILR-detected arrhythmias for whom there is class I indication to cardiac pacing for symptomatic and asymptomatic asystolic pauses or atrioventricular block according to the latest European guidelines (3).

Finally, the estimated benefit of RM we observed may be in part ascribed also to a general improvement of medical care over time that we could not factor in, as our comparison was between two cohorts observed in different years. Our results should be confirmed by randomized studies.

## Conclusions

In our PS-matched controlled study, RM of ILR in patients with unexplained syncope was associated with a 2.5-fold higher chance of evaluations of arrhythmias underlying syncope (>4-fold higher in asymptomatic episodes and 2.8 in arrhythmias justifying pacemaker implantation) as compared to conventional follow-up with biannual in-office visits.

## Data Availability

The raw data supporting the conclusions of this article will be made available by the authors, without undue reservation.
